# Predictors of Mortality in Critically Ill COVID-19 Patients Demanding High Oxygen Flow: A Thin Line between Inflammation, Cytokine Storm, and Coagulopathy

**DOI:** 10.1155/2021/6648199

**Published:** 2021-04-20

**Authors:** Viseslav Popadic, Slobodan Klasnja, Natasa Milic, Nina Rajovic, Aleksandra Aleksic, Marija Milenkovic, Bogdan Crnokrak, Bela Balint, Milena Todorovic-Balint, Davor Mrda, Darko Zdravkovic, Borislav Toskovic, Marija Brankovic, Olivera Markovic, Jelica Bjekic-Macut, Predrag Djuran, Lidija Memon, Milica Brajkovic, Zoran Todorovic, Jovan Hadzi-Djokic, Igor Jovanovic, Dejan Nikolic, Marija Zdravkovic

**Affiliations:** ^1^University Hospital Medical Center, Bezanijska kosa, Belgrade, Serbia; ^2^Institute for Medical Statistics and Informatics, Faculty of Medicine University of Belgrade, Belgrade, Serbia; ^3^Department of Internal Medicine, Division of Nephrology and Hypertension, Mayo Clinic, Rochester, USA; ^4^Clinical Center of Serbia, Belgrade, Serbia; ^5^Faculty of Medicine, University of Belgrade, Belgrade, Serbia; ^6^Institute of Cardiovascular Diseases “Dedinje”, Belgrade, Serbia; ^7^Department of Medical Sciences, Serbian Academy of Sciences and Arts, Serbia; ^8^Clinic for Hematology, Clinical Center of Serbia, Belgrade, Serbia

## Abstract

**Introduction:**

Mortality among critically ill COVID-19 patients remains relatively high despite different potential therapeutic modalities being introduced recently. The treatment of critically ill patients is a challenging task, without identified credible predictors of mortality.

**Methods:**

We performed an analysis of 160 consecutive patients with confirmed COVID-19 infection admitted to the Respiratory Intensive Care Unit between June 23, 2020, and October 2, 2020, in University Hospital Center Bezanijska kosa, Belgrade, Serbia. Patients on invasive, noninvasive ventilation and high flow oxygen therapy with moderate to severe ARDS, according to the Berlin definition of ARDS, were selected for the study. Demographic data, past medical history, laboratory values, and CT severity score were analyzed to identify predictors of mortality. Univariate and multivariate logistic regression models were used to assess potential predictors of mortality in critically ill COVID-19 patients.

**Results:**

The mean patient age was 65.6 years (range, 29–92 years), predominantly men, 68.8%. 107 (66.9%) patients were on invasive mechanical ventilation, 31 (19.3%) on noninvasive, and 22 (13.8%) on high flow oxygen therapy machine. The median total number of ICU days was 10 (25^th^ to 75^th^ percentile: 6–18), while the median total number of hospital stay was 18 (25^th^ to 75^th^ percentile: 12–28). The mortality rate was 60% (96/160). Univariate logistic regression analysis confirmed the significance of age, CRP, and lymphocytes at admission to hospital, serum albumin, D-dimer, and IL-6 at admission to ICU, and CT score. Serum albumin, D-dimer, and IL-6 at admission to ICU were independently associated with mortality in the final multivariate analysis.

**Conclusion:**

In the present study of 160 consecutive critically ill COVID-19 patients with moderate to severe ARDS, IL-6, serum albumin, and D-dimer at admission to ICU, accompanied by chest CT severity score, were marked as independent predictors of mortality.

## 1. Introduction

COVID-19 infection is a pandemic disease that to this date affected more than 70 million people over the world with more than 1.6 million registered death cases [1]. Numerous studies over the last few months have shown a wide specter of clinical presentation of infection with SARS-CoV-2 virus. It is usually presented as bilateral pneumonia, but with multiple extrapulmonary manifestations that can lead to severe complications and death [2–5]. Rapid worsening of clinical status is a result of a combination of severe viral illness, increased demand on the heart, systemic inflammatory response, compounded by low oxygen levels due to pneumonia, and increased propensity for blood clot formation. Supportive oxygenation therapy, as well as mechanical ventilation, is still a key management strategy in treating patients. Although the overall intrahospital mortality is decreasing lately, the mortality rate of patients admitted to the Intensive Care Unit (ICU) remains relatively high [6]. Definitive predictors of poor clinical outcomes are still uncertain, mainly due to the unpredictability of the disease and multiple underlying pathophysiological mechanisms that can affect patient's condition and further prognosis [7–9]. It is shown so far that advanced age, male sex, the presence of comorbidities including hypertension, diabetes mellitus, malignancies, and cardiovascular and cerebrovascular diseases are associated with higher mortality rate [10–12]. Identifying credible predictors of mortality, especially in critically ill patients, is a challenging task considering the variety of different critical conditions, including acute respiratory distress syndrome, secondary infection, shock, acute heart, and kidney injury. Predictors of mortality among laboratory parameters are important as they can reflect possible mechanisms of disease progression and give important information on potentially useful therapeutic modalities [13].

In the present study, we analyzed patients admitted to ICU to evaluate potential independent predictors of mortality in critically ill COVID-19 patients with moderate to severe acute respiratory distress syndrome (ARDS).

## 2. Methods

In this single-center study, we included 160 consecutive patients with confirmed COVID-19 infection admitted to the Respiratory Intensive Care Unit of University Hospital Center Bezanijska kosa, Belgrade, Serbia, between June 23, 2020, and October 2, 2020. University Hospital Center Bezanijska kosa is a reference COVID-19 high-volume center, treating more than 1000 COVID-19 patients per month overall and more than 100 patients in ICU. Infection with SARS-CoV-2 virus was confirmed by real-time PCR assay from a nasopharyngeal swab sample. Chest X-ray was performed at hospital admission and onwards in terms of control if necessary. Chest CT was obligatory at admission, including the estimation of CT severity score. Typical COVID-19 pneumonia changes were evaluated including bilateral lung involvement with ground-glass opacities, consolidations, interlobular septal thickening, and crazy paving patterns, as well as pleural effusion and lymphadenopathy. According to the severity of the observed changes, every lobe was given 0-5 points (for upper, middle, and lower right lung lobe, and upper and lower left lung lobe, respectively) forming the maximum possible score of 25 points. Except scoring, the disease was classified into 4 stages (early, progressive, peak, and absorption). A follow-up chest CT was indicated in case of clinical deterioration and was usually performed right before or after the admission to ICU. Main clinical criteria for Respiratory Intensive Care Unit admission was radiographic or CT scan severity score progression, peripheral oxygen saturation (Sp02) below 93% despite maximal conventional supportive oxygen therapy (up to 15 L/min through a nasal cannula, conventional oxygen, or nonrebreather mask), laboratory test results, mainly an increase of inflammatory parameters after repeated controls and arterial blood gas test. Critically ill patients on invasive, noninvasive ventilation, and high flow oxygen therapy with moderate and severe ARDS were selected for the study according to the Berlin definition of ARDS [14]. Patients on conventional oxygen therapy being admitted to ICU due to gradual clinical deterioration were not included. During the hospitalization, patients were treated according to the adjusted National Protocol of the Republic of Serbia for the treatment of COVID-19 infection. Antiviral agents (favipiravir, remdesivir) were used in 5-7 days from symptom onset in patients on supportive oxygen therapy and with radiographically verified severe bilateral pneumonia. Favipiravir was administered orally, 3200 mg in two doses the first day, and 600 mg in two doses for the next 4 days. Remdesivir was administered intravenously, 200 mg the first day, and 100 mg for the next 4-9 days in consultation with an infectologist. Corticosteroids (prednisone 0.5 mg/kg in two doses, methylprednisolone 1-2 mg/kg, dexamethasone 6 mg/day) were used in patients with moderate to severe clinical image with signs of gradual clinical deterioration or in patients with incipient or developed acute respiratory distress syndrome (ARDS). Anticoagulant therapy was used in standard prophylactic dose of LMWH for patients with multiple risk factors and on conventional oxygen therapy. Therapeutical doses were used, according to the anti-Xa levels, for patients in intensive care unit requiring mechanical ventilation or hi-flow oxygen therapy, those on long-term anticoagulant therapy or in patients with suspectable or confirmed thrombosis. Antibiotics were used empirically or according to the antibiogram. The main criteria for tocilizumab administration was an increase in IL-6 values above 40 ng/L and CRP values above 50 mg/L or a threefold increase during the last 48 h in patients with clinical worsening with more than 25 resp/min, saturation below 93%, and p02 value bellow 8,66 kPa without supportive oxygen therapy. Tocilizumab was administered intravenously at 8 mg/kg body weight (up to a maximum of 800 mg) in two doses, 12 h apart. Convalescent plasma was used in patients with rapid worsening, positive PCR test for SARS-CoV-2 virus, the first two weeks from symptom onset. The recommended dose was 4-5 ml/kg or 200-500 ml per day in two doses. The indication was established according to the specific scoring system with different variables including patient's clinical status, form of the disease, time from symptom onset, respiratory status, radiographic findings, comorbidities, and applied therapy. Among the inotropic agents, noradrenaline, dobutamine, vasopressin, and adrenaline were used. Outcomes were stratified as deceased or discharged from the hospital. All 160 patients were followed until their outcomes.

### 2.1. Data Collection

The data were collected through medical documentation and the hospital's health informational system (Heliant, v7.3, r48602). Demographic data (age, gender, and BMI), past medical history (hypertension, diabetes mellitus, COPD, coronary heart disease, heart failure, and chronic kidney disease), laboratory values (IL-6, CRP, PCT, ferritin, D-dimer, serum albumin, lymphocytes, thrombocytes, prothrombin time, activated partial thromboplastin time, and fibrinogen), and CT severity score were analyzed to identify predictors of mortality in critically ill COVID-19 patients. Clinical and laboratory parameters were followed upon admission to the hospital and Intensive Care Unit, with certain parameters being followed by their peak or lowest values (IL-6, CRP, PCT, D-dimer, serum albumin, lymphocytes, and CT severity score) during hospitalization. IL-6 values were followed in the period before the Tocilizumab administration. Reference values for evaluated laboratory parameters are presented in Supplemental Table 1.

## 3. Results

The study population included 160 consecutive patients admitted to ICU with moderate to severe ARDS due to COVID-19-related pneumonia. The mean patient age was 65.6 years (range, 29–92 years), predominantly men 68.8%. Laboratory values at admission to hospital and ICU are presented in Table 1. D-dimer, CRP, and ferritin levels were elevated, while the absolute number of lymphocytes was decreased. The median CT score was 20 (25^th^ to 75^th^ percentile: 16-23). 107 (66.9%) patients were on invasive mechanical ventilation, 31 (19.3%) on noninvasive, and 22 (13.8%) on high flow oxygen therapy machine. Median total number of ICU days was 10 (25^th^ to 75^th^ percentile: 6-18), while the median total number of hospital stay was 18 (25^th^ to 75^th^ percentile: 12-28). Detailed characteristics for the whole study population, as well as according to the survival groups, are presented in Table 1. The mortality rate was 60% (96/160). Patients who died were older and had higher CRP and D-dimer levels, and lower levels of lymphocytes at admission to hospital, higher CT score, as well as higher levels of D-dimer and IL-6 and lower levels of lymphocytes and serum albumin at admission to ICU, and they were significantly more on mechanical ventilation. There were no differences in the number of days from the beginning of symptoms to hospital admission or ICU admission between deceased and discharged patients. Tocilizumab was administered to 38 patients (23.8%). There were no differences in mortality between the groups according to Tocilizumab use. In addition, there were no significant baseline differences between the patients who received and did not receive Tocilizumab in the ICU, other than for age, i.e., patients who received Tocilizumab in the ICU were younger (see Supplemental Table 2).

The presence of comorbidities is presented in Table 2. There were 37 patients without comorbidities (23.7%) and 48 with one comorbidity (30.8%), while 71 patient had multiple comorbidities (45.5%). The total number of patients with comorbidities was 120 (76.4%). There were no significant differences in the presence of comorbidities according to the survival groups.

Univariate logistic regression analysis confirmed the significance of the following variables associated with the mortality of patients admitted to ICU due to COVID-19-related pneumonia: age, CRP, and lymphocytes at admission to hospital, albumin, D-dimer, and IL-6 at admission to ICU, and CT score Table 3.

**Figure 1:**
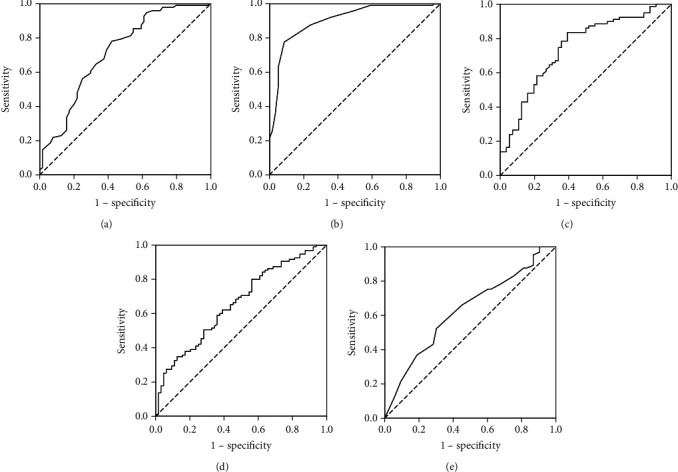
ROC curves for (a) age, (b) serum albumin, (c) IL-6, (d) D-dimer, and (e) CT score.

Based on ROC curves (Figure 1) we determined cut-off points for significant continuous variables from the logistic regression analysis predicting mortality and used the categorical variables further in analysis to develop easy to use predictive model.

The following variables were independently associated with mortality in the final multivariate analysis: serum albumin, IL-6, and D-dimer at admission to ICU (Table 4).

## 4. Discussion

The present study on critically ill COVID-19 patients with moderate to severe ARDS showed a correlation of serum albumin, IL-6, and D-dimer all together at admission to ICU, accompanied by chest CT severity score, as independent predictors of mortality. These results are relying on the fact that cytokine storm and endothelial injury with induced procoagulable state have been marked as essential pathophysiological mechanisms of multiorgan failure and death in patients with severe COVID-19 infection [15–20].

It is important to point out that the results from univariate logistic regression analysis revealed age, CRP, and lymphocytes at admission to hospital as predictors of mortality in patients admitted to ICU, while serum albumin, D-dimer, IL-6, and CT severity score were significant predictors of mortality at admission to the ICU. Final multivariate analysis revealed serum albumin, IL-6, and D-dimer at admission to ICU as independently associated with mortality. These results are reflecting the unpredictability of the disease and its clinical course, aggravating the usage of appropriate therapy in a timely manner.

Hypoalbuminemia (serum albumin levels below 35 g/L) is more severe in critically ill patients and is associated with poor outcomes [21]. Its correlation with elevated D-dimer values in these patients, as in the study by Violi et al., has been linked to an enhanced risk of arterial and venous thrombosis [22]. Patients showed not only higher levels of D-dimer but also higher levels of CRP and creatinine, probably due to increased vascular permeability, kidney, or liver disease. Serum albumin is also a marker of severe oxidative stress and an acute phase reactant with antioxidant properties that may undergo irreversible oxidation [21]. It is a source of free thiols that can expel reactive oxidant species. Reactive oxidant species comprehend platelet and clotting activation, which is the reason why more patients with severe hypoalbuminemia are at greater risk of potential thrombotic events. Overall, synthesis in the liver is downregulated due to the effects of cytokines being released during the cytokine storm. Hypoalbuminemia followed by massive fluid loss due to severe infection is also responsible for hypovolemia and shock in these patients [23]. Correlation of albumin levels and severity of the infection is presented through the specific CRP/albumin ratio, already marked as an independent risk factor for severe COVID-19 infection [24, 25].

IL-6 induces oxidative stress and endothelial dysfunction by overexpression of the Angiotensin II type 1 receptor, which was presented 15 years ago in paperwork by Wassman et al. [26]. This also represents an important pathogenetic mechanism of the atherosclerotic process. Having in mind the cell entry mechanism of the SARS-CoV-2 virus through ACE2 receptors, the viral invasion is subsequently causing a dysregulation between ACE, Angiotensin II, and AT1 receptors, favoring the progression of inflammatory and thrombotic processes [27]. Lately, several studies have presented IL-6 as an independent predictor of patient outcomes in terms of the severity of the disease and survival [28, 29]. In our study, the values of IL-6 during hospitalization were followed before tocilizumab administration, as there was an elevation of IL-6 levels after usage due to disrupted clearance after drug saturation of the receptors. In a large study evaluating the role of different cytokines in COVID-19 infection, only IL-6 and TNF-*α* showed significant prognostic value [28]. Unlike other states where cytokine levels are increased, as in sepsis, the levels of IL-6 and TNF-*α* in patients with COVID-19 were sustained during days or even weeks, which makes the decision for administering the anticytokine treatment on time more difficult. Evaluating the levels of IL-6 early in disease onset can stratify patients at higher risk to develop a more severe form of the disease [30].

Increased levels of D-dimer, a fibrin degradation product, are associated with worse prognosis in patients with COVID-19, including an increased risk of ICU admission, mechanical ventilation, and death [31–33]. Severe endothelial dysfunction, subsequently caused by direct viral involvement and overproduction of cytokines, is causing a hypercoagulable state creating a suitable soil for intravascular thrombosis in both macro and microcirculation. The incidence of pulmonary embolism has been increased in patients with COVID-19 infection, estimated to be up to 9% of the cases, verified by CT pulmonary angiography [34]. In certain studies, venous thrombosis was found in up to 35% of the cases, mainly as deep vein thrombosis (DVT) [35]. Patients with ARDS within COVID-19 infection have a substantially increased rate of pulmonary embolism, estimated to be 11.8%, while patients diagnosed with thrombotic complications have more than a 5-fold increase in all-cause mortality [36]. The reported percentage of patients with increased D-dimer levels in ICU is going up to 81%, speaking in favor of a large portion of patients with microthrombosis as the main presentation of hypercoagulable state [37]. The overall coagulation status in patients with COVID-19 is extremely significant, as prolonged prothrombin time and activated partial thromboplastin time are also found to be associated with higher mortality from COVID-19 infection [38, 39].

Chest CT severity score is a useful imaging modality tool in evaluating the extensiveness of pulmonary involvement and stratifying patients not only numerically but also descriptively by estimating in which of the 4 stadiums of the disease the patient is (early, progressive, peak, or absorption) [40, 41]. In our study, the cut-off value of 20 points was related to poor clinical outcome, according to the prognostic logistic model. Previous studies on the importance of chest CT severity score are supporting this conclusion, with similar cut-off values being defined [42].

During the hospitalization, patients were strictly treated according to the protocol, as explained in the Methods section. Previously, the effects of anticoagulant therapy have been undisputedly proven to be connected with improved survival. In hospitalized COVID-19 patients receiving both prophylactic and therapeutical doses, there is a 30% lower chance of intubation and 50% higher chance of survival, especially in patients receiving low molecular weight heparin, as used in our study [43]. Although the anticoagulant therapy in COVID-19 patients has become almost mandatory in treatment protocols, the significant decrease in overall mortality of critically ill patients has not been noted yet, speaking in favor of multiple pathophysiological mechanisms being responsible for poor clinical outcomes.

The main difference between used therapeutic modalities in our study was the introduction of antiviral agents (remdesivir and favipiravir), although only 19 (11.8%) patients did not have it available during hospitalization. Considering the effects on outcomes, WHO recently recommended against the use of remdesivir, as evidence suggested no effects on mortality, need for mechanical ventilation, and other outcomes [44]. The positive effects, as presented by Beigel et al., are for now limited to evidence of shorter time to recovery, as this was the primary endpoint of the study [45]. Favipiravir promotes rapid viral clearance and a higher clinical recovery rate by shortening the disease course and reducing the need for oxygen requirement. However, the supportive data on resulting in lower rates of respiratory failure, ICU admissions, or all-cause mortality is missing [46]. Further studies will provide more insights into its clinical safety and efficacy.

Potential therapeutic effects of albumins must be considered, having in mind that serum albumin was the strongest independent predictor of mortality in our study. It is already shown that the administration of albumin in critically ill patients with acute respiratory distress syndrome might have its advantages. It improves oxygenation early after the treatment, probably by reducing the alveolar-capillary leakage, but without effects on the overall mortality rate [47]. Several limitations regarding the administration of albumin solutions are inevitable, including dosage, treatment length, possible effects on renal function, and the optimal moment of therapy initiation. However, the usefulness in critically ill COVID-19 patients is yet to be established in randomized controlled trials.

Speaking of novel therapeutic modalities, it is important to emphasize that in our results, there were no differences in mortality between the groups according to Tocilizumab use. To properly estimate the effectiveness of Tocilizumab and the possible role of IL-6 as an independent predictor of mortality, patients that received Tocilizumab before admission to ICU were not included in the study. Also, the baseline characteristics between the groups were not statistically significant, which is especially important to underline (Supplemental Table 2). After numerous trials investigating the potential benefits of this IL-6 receptor blocker, the definitive results are still controversial, probably due to an undetermined time frame of the best usability in correlation with clinical course [48, 49].

There are several limitations to the study. This is a single-center study. Although the sample size is quite satisfying, considering that these are critically ill patients, future multicenter, prospective studies will shed light on stronger correlations between different predictors of mortality in COVID-19 patients paving the way for potentially useful novel therapeutic modalities. ICU scoring systems (mainly SOFA and APACHE II scores) were not used as a part of the data considering their clinical utility has already been proven in several studies [50, 51].

## 5. Conclusion

In this single-center study of 160 consecutive critically ill COVID-19 patients with moderate to severe ARDS demanding high oxygen flow, serum albumin, IL-6, and D-dimer at admission to ICU, accompanied by a chest CT severity score, were marked as independent predictors of mortality. This conclusion supports previous studies on cytokine storm and diffuse microvascular thrombosis/thrombotic events as potential mechanisms of poor clinical outcomes. Further larger prospective multicenter studies are necessary to determine the exact correlation between different predictors of mortality in order to stratify patients with a significant chance of developing a severe form of the disease.

## Figures and Tables

**Table 1:** Characteristics of patients admitted to ICU with moderate to severe ARDS due to COVID-19-related pneumonia, overall and subpopulation analysis according to survival.

	Total (*n* = 160)	ICU patients		*p*
		Live (*n* = 64)	Dead (*n* = 96)	
Gender, *n* (%)				
Male	110 (68.8)	47 (73.4)	63 (65.6)	0.384
Female	50 (31.3)	17 (26.6)	33 (34.4)	
Age, mean ± sd	65.6 ± 14	58.8 ± 15.1	70.2 ± 11.1	**<0.001**
Laboratory at admission to hospital, median (IQR)				
CRP	81.2 (53.8–173.4)	74.7 (46.7–117.5)	105.2 (58.1–200.5)	**0.008**
Lymphocytes	0.74 (0.53–1.04)	0.84 (0.56–1.36)	0.66 (0.46–0.86)	**0.001**
D-dimer	881 (523–2660)	646 (372–1582)	1051 (650–3642)	**0.004**
Ferritin	875 (497–1482)	850 (516–1521)	891 (484–1482)	0.794
Thrombocytes	235 (177–329)	247 (200–345)	228 (159–302)	0.096
INR	1.13 (1.01–1.30)	1.12 (0.99–1.28)	1.14 (1.04–1.34)	0.130
PT	87 (69–100)	90 (72–103)	86 (68–99)	0.325
APTT	25.2 (21.9–29–8)	24.1 (21.4–28.0)	26.1 (22.7–30.2)	0.077
Fibrinogen	3.9 (3.4–4.9)	4.0 (3.5–4.8)	3.8 (3.2–4.9)	0.605
Laboratory at admission to ICU, median (IQR)				
CRP	87.8 (51.5–173.3)	78.4 (52.3–148.9)	95.1 (50.7–195.9)	0.166
Lymphocytes	0.63 (0.44–0.88)	0.7 (0.49–0.99)	0.61 (0.38–0.81)	**0.019**
D-dimer	1414 (701–5030)	1027 (566–2585)	1944 (860–7423)	**0.001**
Ferritin	1108 (485–1698)	1181 (625–1640)	1058 (454–1764)	0.947
Thrombocytes	191 (150–278)	191 (151–259)	195 (149–291)	0.668
Serum albumin	31 (29–34)	35 (33–37)	29 (27–31)	**<0.001**
INR	1.15 (1.05–1.29)	1.13 (1.01–1.28)	1.16 (1.08–1.35)	0.087
PT	89 (72–103)	96 (74–108)	86 (69–99)	0.115
APTT	25.9 (23.1–28.5)	25.4 (22.7–27.6)	26.1 (23.3–29.8)	0.272
Fibrinogen	4.3 (3.6–5.3)	4.4 (3.7–5.4)	4.2 (3.6–5.2)	0.744
IL6	91 (38.80–286.00)	50.42 (24.45–100.90)	133.80 (74.00–426.80)	**<0.001**
CT score	20 (16–23)	19 (16–22)	21 (19–23)	**0.017**
From beginning of symptoms to hospital admission (days), median (IQR)	7 (4–9)	7 (5–9)	7 (4–10)	0.844
From beginning of symptoms to IUC admission (days), median (IQR)	10 (8–12)	11 (8–13)	10 (7–12)	0.461
Mechanical ventilation, *n* (%)	107 (66.9)	13 (20.3)	94 (97.9)	**<0.001**
Tocilizumab, *n* (%)	38 (23.8)	13 (20.3)	25 (26)	0.452

**Table 2:** The presence of comorbidities in the study population.

Comorbidities, *n* (%)	Total (*n* = 160)	ICU patients		*p*
		Live (*n* = 64)	Dead (*n* = 96)	
Hypertension	109 (69.4)	40 (62.5)	69 (74.2)	0.158
Diabetes	52 (33.1)	26 (40.6)	26 (28.0)	0.121
Obesity	14 (8.9)	6 (9.5)	8 (8.5)	1.000
HOBP	8 (5.1)	1 (1.6)	7 (7.5)	0.095
Asthma	6 (3.8)	2 (3.1)	4 (4.3)	0.706
Coronary disease	28 (17.8)	10 (15.6)	18 (19.4)	0.672
Cardiomyopathy	14 (8.9)	6 (9.4)	8 (8.6)	1.000
Number of comorbidities				
None	37 (23.7%)	18 (28.6%)	19 (20.4%)	0.457
1	48 (30.8%)	17 (27.0%)	31 (33.3%)	
≥2	71 (45.5%)	28 (44.4%)	43 (46.2%)	
Total number of patients with comorbidities	120 (76.4%)	46 (71.9%)	74 (79.6%)	0.339

**Table 3:** Univariate logistic regression analysis for mortality of patients admitted to ICU due to COVID-19-related pneumonia, continuous variables used in the model.

Variable	*p*	RR	95% CI for RR
Age	<0.001	1.067	1.039–1.097
At admission to hospital			
CRP	0.013	1.005	1.001–1.009
Lymphocytes	0.003	0.341	0.168–0.690
D-dimer	0.585	1.000	1.000–1.000
At admission to ICU			
Lymphocytes	0.059	0.513	0.256–1.027
D-dimer	0.013	1.000	1.000–1.000
Serum albumin	<0.001	0.553	0.455–0.673
IL6	0.020	1.002	1.000–1.003
CT score	0.032	1.089	1.008–1.178
Mechanical ventilation	<0.001	184.385	40.037–849.161

**Table 4:** The univariate logistic regression analysis with categorical variables used and the full multivariate prognostic logistic model predicting mortality.

Variable	Univariate analysis			Multivariate analysis		
	*p*	RR	95% CI for RR	*p*	RR	95% CI for RR
Age > 65 yrs	<0.001	3.495	1.801–6.779	/	/	/
At admission to ICU						
Albumin < 33	<0.001	22.286	9.319–53.294	<0.001	25.740	7.491–88.443
IL–6 > 72	<0.001	6.100	2.857–13.023	0.002	6.245	1.937–20.129
D − dimer > 1000	0.026	2.111	1.091–4.085	0.013	4.574	1.375–15.212
CT score ≥ 20	0.024	2.362	1.120–4.980	/	/	/

## Data Availability

The data that support the findings of this study are available from the corresponding author (MZ) upon reasonable request.

## Abbreviations

COVID-19: Coronavirus disease 19
ICU: Intensive Care Unit
ARDS: Acute Respiratory Distress Syndrome
CT: Computerized tomography
CRP: C-reactive protein
CAR: CRP/albumin ratio
IL-6: Interleukin-6
SARS-CoV-2: Severe acute respiratory syndrome, coronavirus 2
PCR: Polymerase chain reaction
LMWH: Low-molecular-weight heparin
COPD: Chronic obstructive pulmonary disease
PCT: Procalcitonin
ACE-2: Angiotensin-converting enzyme 2
TNF-*α*: Tumor necrosis factor-*α*
APACHE-II: Acute Physiology and Chronic Health Evaluation
SOFA: Sequential Organ Failure Assessment.

## Supplementary Materials

Supplementary materials Supplemental Table 1: reference values of evaluated laboratory parameters. Supplemental Table 2: baseline differences between the patients who received and did not receive Tocilizumab in the ICU.
