# Supplementary material for: Predictors of Mortality in Critically Ill COVID-19 Patients Demanding High Oxygen Flow: A Thin Line between Inflammation, Cytokine Storm, and Coagulopathy
Source: Oxid Med Cell Longev. 2021 Apr 20;2021:6648199. doi: 10.1155/2021/6648199 (PMC8081622; doi:10.1155/2021/6648199)
Supplement: Supplementary materials — Supplemental Table 1: reference values of evaluated laboratory parameters. Supplemental Table 2: baseline differences between the patients who received and did not receive Tocilizumab in the ICU. [file 6648199.f1.docx]

**Supplemental Table 1.**

| **Reference values of laboratory parameters** | |
| --- | --- |
| CRP | 0.0-5.0 mg/L |
| Lymphocytes | 1.0-4.0 x10^9^/L |
| D-dimer | 0.0-500.0 ng/mL |
| Ferritin | 30.0-400 ug/L |
| Thrombocytes | 150.0-450.0 x10^9^/L |
| Serum albumin | 35.0-52.0 g/L |
| INR | 0.9-1.1 |
| PT | 82.0-121.0 % |
| APTT | 23.0-31.9 s |
| Fibrinogen | 2.1-4.0 g/L |
| IL6 | 0.0-7.0 pg/mL |
| PCT | 0.00-0.05 ng/mL |

**Supplemental Table 2.**

|  | **Tocilizumab use** | | **p** |
| --- | --- | --- | --- |
|  | **Yes (n=38)** | **No (n=122)** |  |
| **Gender, n (%)** |  |  |  |
| Male | 30 (78.9) | 80 (65.6) | 0.161 |
| Female | 8 (21.1) | 42 (34.4) |  |
| **Age, mean±sd** | 60.0±12.0 | 67.0±14.0 | **0.001** |
| **Laboratory at admission, median (IQR)** |  |  |  |
| CRP | 89.3(53.7–184.9) | 81.2 (53.9–169.5) | 0.568 |
| Lymphocytes | 0.71 (0.55–1.06) | 0.74 (0.49–1.01) | 0.797 |
| D–dimer | 735 (457–1412) | 975 (549–3455) | 0.166 |
| Ferritin | 1253 (757–1729) | 828 (467–1400) | **0.031** |
| Thrombocytes | 257 (168–339) | 233 (179–320) | 0.765 |
| INR | 1.10(0.98–1.23) | 1.14 (1.04–1.33) | 0.087 |
| PT | 94 (77–101) | 83 (68–100) | 0.065 |
| APTT | 23.6 (21.3–26.6) | 25.9 (22.4–30.1) | 0.077 |
| Fibrinogen | 4.0 (3.5–5.0) | 3.9 (3.3–4.7) | 0.491 |
| **Baseline laboratory, median (IQR)** |  |  |  |
| CRP | 92.5 (62.3–188.5) | 85.3(49.9–166.0) | 0.293 |
| Lymphocytes | 0.55 (0.40–0.83) | 0.65 (0.46–0.89) | 0.216 |
| D–dimer | 1260 (769–4030) | 1437 (696–5129) | 0.854 |
| Ferritin | 1415 (998–1915) | 916 (444–1598) | **0.003** |
| Thrombocytes | 183 (141–226) | 194 (151–292) | 0.150 |
| Serum albumin | 32(29–34) | 31 (29–34) | 0.936 |
| INR | 1.13 (1.02–1.25) | 1.17 (1.05–1.32) | 0.344 |
| PT | 93 (77–105) | 88 (71–100) | 0.301 |
| APTT | 25.7 (23.7–26.7) | 25.9 (22.7–29.3) | 0.469 |
| Fibrinogen | 4.6 (3.9–5.9) | 4.2 (3.6–5.2) | 0.105 |
| CAR | 3.03 (1.95–6.23) | 2.32(1.59–5.09) | 0.181 |
| **CT score** | 21 (19–24) | 20 (16–23) | 0.056 |
| **From beginning of symptoms to hospital admission (days), median (IQR)** | 7 (6–10) | 7 (4–9) | 0.087 |
| **From beginning of symptoms to IUC admission (days), median (IQR)** | 10 (8–12) | 10 (8–13) | 0.324 |
| **Mechanical ventilation, n (%)** | 26 (68.4) | 81 (66.4) | 1.000 |
| **Comorbidities, n (%)** | 31 (81.6) | 89 (74.8) | 0.511 |
| Hypertension | 28 (73.7) | 81 (68.1) | 0.551 |
| Diabetes | 15 (39.5) | 37 (31.1) | 0.429 |
| Obesity | 3 (7.9) | 11 (9.2) | 1.000 |
| HOBP | 1 (2.6) | 7 (5.9) | 0.681 |
| Asthma | 0 (0.0) | 6 (5.0) | 0.158 |
| Coronary disease | 6 (15.8) | 22 (18.5) | 0.811 |
| Cardiomyopathy | 1 (2.6) | 13 (10.9) | 0.190 |
